# Real-world data on the abuse potential of medications for the treatment of insomnia: a disproportionality analysis of the FAERS database

**DOI:** 10.3389/fphar.2025.1735180

**Published:** 2026-01-28

**Authors:** Paul Saskin, William V. McCall, David N. Neubauer, Antonio Crucitti, Bradford Perry, Pierre Philippe Luyet, Riphed Jaziri, Cedric Vaillant

**Affiliations:** 1 Idorsia Pharmaceuticals US Inc, Radnor, PA, United States; 2 Department of Psychiatry and Health Behavior, Medical College of Georgia at Augusta University, Augusta, GA, United States; 3 Department of Psychiatry and Behavioral Sciences, School of Medicine, Johns Hopkins University, Baltimore, MD, United States; 4 Idorsia Pharmaceuticals, Allschwil, Switzerland

**Keywords:** drug abuse, dual orexin receptor antagonists, FDA adverse event reporting system, insomnia, schedule IV drugs

## Abstract

**Background:**

Insomnia disorder is a chronic medical condition estimated to affect 12% of adults. The potential for abuse of hypnotics often contributes to physician reluctance to prescribe medications to treat insomnia as a chronic condition. This study examined the real-world abuse potential of approved and off-label medications used to treat insomnia, employing data from the FDA Adverse Event Reporting System (FAERS) database.

**Methods:**

Data from 1 January 2014 to 31 March 2024 were retrieved. Drugs of interest included Schedule IV drugs (benzodiazepines, Z-drugs, dual orexin receptor antagonists [DORAs]) and non-scheduled drugs (trazodone, doxepin, ramelteon). Relevant reported adverse events denoting drug abuse were identified if they contained an event with any preferred terms from the SMQ Drug abuse, dependence, and withdrawal (MedDRA v26.1), with cases of overdose due to suicide attempts excluded. The reporting odds ratios (ROR) and proportional reporting ratios (PRR) were used as disproportionality measures.

**Results:**

Rates of adverse event cases of abuse, dependence, and withdrawal retrieved were highest for benzodiazepines approved for any indication, followed by benzodiazepines approved for insomnia, trazodone, doxepin, Z-drugs, ramelteon, and DORAs. DORAs were associated with a low ROR value relative to Z-drugs (ROR = 0.150; 95% CI [0.131, 0.171]) and to trazodone (ROR = 0.092; 95% CI [0.081, 0.105]). Similar results were obtained using the PRR. The DORA class had the lowest rates of adverse event denoting drug abuse, even lower than the unscheduled drugs ramelteon and doxepin, which are known not to be prone to abuse or dependence. Furthermore, the DORA class had significantly lower odds of reporting for adverse events denoting drug abuse when compared with zolpidem or the unscheduled medication trazodone.

**Conclusion:**

This study identified significantly fewer reported cases of real-world abuse, misuse, overdose, and other safety risks for DORAs compared with the unscheduled drug trazodone and scheduled Z-drugs. This suggests that categorization of DORAs as Schedule IV drugs may overstate their abuse potential.

## 1 Introduction

Insomnia disorder is a chronic medical condition characterized by difficulty initiating and/or maintaining sleep, early morning awakenings, and impaired daytime functioning (Riemann et al., 2022; APA, 2022). Chronic insomnia can exacerbate or be associated with an increased risk of psychiatric diseases, such as anxiety, depression, and suicide, and can adversely affect physical health, increasing the risk of cardiovascular disease, diabetes, and other conditions (Chasens et al., 2021; Kwok et al., 2018; Wu et al., 2023; McCall and Black, 2013). Effective treatment that normalizes sleep over a prolonged period is essential for individuals with insomnia disorder.

Most medical societies have identified cognitive behavior therapy for insomnia (CBTi) as the first-line treatment for insomnia disorder. CBTi employs standard sleep hygiene recommendations (stabilization of a sleep-wake routine and improving the sleep environment) and non-pharmacologic strategies (stimulus control and sleep restriction) to alleviate symptoms with fewer overall side effects than most pharmacologic interventions. The widespread application of CBTi is limited by the need for active cooperation from patients and restricted access to CBTi-trained sleep specialists (Riemann et al., 2023; Schutte-Rodin et al., 2008; Thomas et al., 2016). Pharmacologic treatment is often used in patients who experience chronic insomnia, especially if CBTi is ineffective or not available (Riemann et al., 2023; Sateia et al., 2017).

Physician concerns over the use of sedative-hypnotics have been present for decades. Much of this has been due to the limited data available on long-term use of medications to promote sleep despite them routinely being used for extended periods of time. A recent survey of physicians who routinely treat insomnia disorder suggested that 50% expressed concern about long-term use of medications for insomnia therapy (Zee et al., 2023). The safety profiles of insomnia-specific medications have improved as a result of ongoing clinical development. Recent clinical trials have demonstrated efficacy and safety of several insomnia drugs with most recent trials examining these parameters for periods of up to 1 year of nightly use (Michelson et al., 2014; Kärppä et al., 2020; Mignot et al., 2022).

The United States (US) Food and Drug Administration (FDA)-approved medications for the management of insomnia include benzodiazepines, non-benzodiazepine receptor agonists (also known as Z-drugs), melatonin receptor agonists, low-dose doxepin, and, most recently, dual orexin receptor antagonists (DORAs). DORAs differ from other insomnia medications in their mechanism of action. Benzodiazepines and Z-drugs primarily induce a sedative and hypnotic effect by enhancing the effect of GABA neurotransmitters (Madari et al., 2021; Rosenberg et al., 2023). Doxepin has multiple effects but works primarily as an antagonist of H1 receptors. Ramelteon is a specific agonist of both melatonin receptors (MT1, MT2) and has its purported effect by enhancing melatonin signaling (Miyamoto, 2009). The mechanism of the DORA medications is through a reduction of arousal via antagonism of both orexin receptors, selectively suppressing the overactive wake drive, which can disrupt sleep onset and maintenance (Muehlan et al., 2023). DORA medications are less prone to dependency and tolerance and have fewer side effects than other insomnia drugs (Rosenberg et al., 2023; Chakraborty et al., 2023).

Three DORAs are approved in the US for the treatment of insomnia characterized by difficulties with sleep onset and/or sleep maintenance: suvorexant (approved in 2014), lemborexant (approved in 2019), and daridorexant (approved in 2022). Despite their well-demonstrated clinical safety from registrational trials and non-clinical data showing a distinct profile with limited evidence of abuse potential or drug withdrawal (Mignot et al., 2022; Rosenberg et al., 2019; Herring et al., 2012; Michelson et al., 2014; Asakura et al., 2021 Steiner et al., 2023), DORAs were classified by the US Drug Enforcement Administration (DEA) as Schedule IV medications, similar to benzodiazepines and Z-drugs (i.e., drug products with a low potential for abuse, and low risk of dependence) (United States Drug Enforcement Administration, 2024). Well-established animal models and safety data from large randomized clinical trials did not reveal any specific risks of abuse potential and dependence associated with the DORA class. The placement of DORAs into Schedule IV was based on the results of Human Abuse Potential (HAP) studies, which assess “likability” of a drug in individuals with a history of recreational use of sedative drugs but without underlying features of insomnia or disturbed sleep (Landry et al., 2022; Schoedel et al., 2016; Ufer et al., 2022).

Abuse potential of drugs with central nervous system activity is a critical component of the development of new medications for regulatory filings and approval. A recent expert panel of the International Study Group Investigating Drugs as Reinforcers (ISGIDAR) suggested that traditional HAP Studies as outlined in the 2017 FDA Guidance to Industry (United States Food and Drug Administration, 2017) may not be entirely appropriate or accurate for drugs with novel mechanisms of action that have not shown evidence of abuse potential in pre-clinical and clinical studies (Henningfield et al., 2025). The FDA’s Controlled Substance Staff have proposed that HAP studies have increasingly emerged as a discrepant model that overestimates abuse potential and risk of post-marketing abuse (Calderon et al., 2020). They found that the DORA class of medications would benefit from a better understanding of the actual rates of abuse in the real-world setting (Caro et al., 2022).

Several recent studies have performed an analysis of the FDA Adverse Event Reporting System (FAERS) assessing the reported adverse event profile of the DORA class (Cicala et al., 2024; Kadyala et al., 2025; Wang et al., 2024; Jiang et al., 2024). These studies have used a similar methodology, and all identified global categories of adverse events. However, they were not specifically designed to capture the abuse related potential of the DORA class relative to the other medications prescribed for the treatment of insomnia. The present analysis was conducted to provide a real-world context of the abuse potential of medications used for insomnia, from the FAERS database spanning across 10 years of pharmacovigilance experience. To facilitate the interpretation of the results, a disproportionality analysis was implemented to characterize the abuse potential of the DORA class among the most widely prescribed drugs for insomnia in the US, using the Schedule IV drug zolpidem as the most widely prescribed member of the Z-drug class and the unscheduled drug trazodone as reference drugs. Trazodone was selected as it has become one of the most widely prescribed drugs for the treatment of insomnia in the US, with over 85% of trazodone prescriptions associated with a diagnosis of insomnia (McIntyre et al., 2025; Kadyala et al., 2025, Wong et al., 2020). This is despite trazodone not being FDA approved for this condition and against the recommendations of major medical societies (Sateia et al., 2017).

This analysis attempts to provide a focused look at the key characteristics of insomnia medications that are of paramount concern to patients and prescribing providers, those associated with abuse, dependence, and withdrawal.

## 2 Materials and methods

The FAERS database is publicly accessible and widely utilized by external customers, including researchers, institutions, and Marketing Authorization Holders (Khaleel et al., 2022; FAERS, 2024). It contains information on post-marketing self-reported adverse events and medication error reports submitted to the FDA and supports the FDA’s post-marketing safety surveillance program for drug and therapeutic biologic products. The informatic structure of the FAERS database adheres to the international safety reporting guidance issued by the ICH E2B and uses Medical Dictionary for Regulatory Activities (MedDRA) terminology (MedDRA, 2023).

As the main objective of this study was to examine the real-world abuse potential of the DORA class using a comprehensive characterization of FAERS data, we compared the occurrence of unsolicited reported drug abuse events defined via Standardized MedDRA Queries (SMQ). Drugs of interest were those approved by the FDA for the treatment of insomnia, with Schedule IV drugs and non-scheduled drugs used in or approved for insomnia considered. Among the Schedule IV drugs, benzodiazepines (estazolam, flurazepam, quazepam, temazepam, triazolam), Z-drugs (zolpidem, zaleplon, eszopiclone), and DORAs (suvorexant, lemborexant, daridorexant) were selected. The non-scheduled drugs included trazodone (frequently prescribed for insomnia in the US, albeit not FDA-approved for insomnia (Sateia et al., 2017)), doxepin, and ramelteon.

### 2.1 Identification of adverse events denoting drug abuse

The FAERS database was queried to identify all cases of adverse events for the drugs of interest if they were reported in the US (i.e., the country where the event occurred) from 1 January 2014 to 31 March 2024. Duplicate cases were removed, and counts were calculated with the number of cases based on the unique ID provided by the FAERS database.

Relevant reported adverse events denoting drug abuse were then identified following a 2-step approach. First, cases were included if they contained an event with any preferred terms (PTs) from the SMQ Drug abuse, dependence, and withdrawal (MedDRA v26.1) (see Table 1 for the complete list of PTs). Second, cases were excluded if they reported the PTs “Accidental overdose” OR “Intentional overdose” OR “Overdose” OR “Prescribed overdose” OR “Toxicity to various agents” (without any other PTs from the SMQ Drug abuse, dependence, and withdrawal) AND any event of suicidal behavior (defined by the PTs “Completed suicide” OR “Suicidal behavior” OR “Suicide attempt” OR “Suicide threat” OR “Suspected suicide” OR “Suspected suicide attempt”). This exclusion criterion was implemented to remove cases of overdose due to suicide attempts, as these cases would not qualify for drug abuse. This resulted in the modified SMQ Drug abuse, dependence, and withdrawal.

**TABLE 1:** Preferred terms from the SMQ Drug abuse, dependence, and withdrawal (MedDRA v26.1 [29]).

Preferred terms	​	​
• Accidental overdose	• Antidepressant discontinuation syndrome	• Caffeine dependence
• Cannabinoid hyperemesis syndrome	• Cholinergic rebound syndrome	• Delusion of parasitosis
• Dependence	• Disturbance in social behavior	• Dopamine agonist withdrawal syndrome
• Dopamine dysregulation syndrome	• Drug abuse	• Drug abuser
• Drug dependence	• Drug dependence, antepartum	• Drug dependence, postpartum
• Drug detoxification	• Drug diversion	• Drug level above therapeutic
• Drug level increased	• Drug rehabilitation	• Drug screen
• Drug screen positive	• Drug tolerance	• Drug tolerance decreased
• Drug tolerance increased	• Drug use disorder	• Drug use disorder, antepartum
• Drug use disorder, postpartum	• Drug withdrawal convulsions	• Drug withdrawal headache
• Drug withdrawal maintenance therapy	• Drug withdrawal syndrome	• Drug withdrawal syndrome neonatal
• Incorrect route of product administration	• Intentional device misuse	• Intentional overdose
• Intentional product misuse	• Intentional product use issue	• Maternal use of illicit drugs
• Medication overuse headache	• Multiple use of single-use product	• Narcotic bowel syndrome
• Needle track marks	• Neonatal complications of substance abuse	• Overdose
• Performance enhancing product use	• Pharmaceutical nomadism	• Prescription drug used without a prescription
• Prescription form tampering	• Product administered at inappropriate site	• Rebound effect
• Reversal of opiate activity	• Steroid withdrawal syndrome	• Substance abuse
• Substance abuser	• Substance dependence	• Substance use
• Substance use disorder	• Substance-induced mood disorder	• Substance-induced psychotic disorder
• Topical steroid withdrawal reaction	• Toxicity to various agents	• Withdrawal arrhythmia
• Withdrawal catatonia	• Withdrawal syndrome	​

MedDRA, medical dictionary for regulatory activities; SMQ, Standardized MedDRA, queries.

### 2.2 Data analyses

#### 2.2.1 Descriptive analysis

The number and percentage of adverse event cases (i.e., events of interest) spontaneously reported for each specific drug were computed for all PTs and the overall modified SMQ. The total number of cases linked to each drug was used to calculate the percentage of cases denoting drug abuse. Only PTs included in the modified SMQ with a frequency threshold ≥1% in any drug group and the overall modified SMQ are reported.

#### 2.2.2 Disproportionality analysis

Two measures of disproportionality (i.e., reporting odds ratios [ROR] and proportional reporting ratios [PRR]) for signal detection were selected to improve sensitivity, specificity, and predictive value. Each method has advantages and disadvantages and can complement the other (Chen et al., 2024).

The ROR and PRR were used as disproportionality measures to evaluate whether there was a greater or lower frequency of reporting of adverse event cases compared with the reference drugs. The ROR compared the odds of reporting an event of interest (from the total number of cases) for a particular drug to the odds of reporting the same event for the reference drug. The PRR is the ratio of the proportion of reports of an event of interest among the total number of cases linked to a particular drug to the corresponding proportion for the comparator drug.

RORs and PRRs were computed for each PT and the overall modified SMQ. Values <1 indicated a lower reporting rate of the event of interest than the reference drug, with no significant difference between a particular drug and the reference drug if 1 was included in the 95% CI limits.

Zolpidem and trazodone were used as reference drugs for Schedule IV and unscheduled drugs, respectively. As benzodiazepines may be used for different indications and to address the potential indication bias in the FAERS database, benzodiazepines approved for any indication and those authorized for insomnia were included and analyzed separately.

All statistical analyses were performed using SAS (version 9.4, SAS Studio, Cary, NC, USA). To ensure methodological transparency and reproducibility, this study adhered to the READUS-PV guideline.

## 3 Results

**FIGURE 1:**
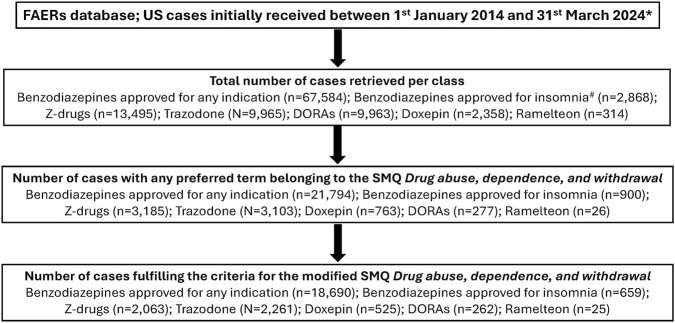
US cases retrieved from the FAERS by steps of data extraction: overall, SMQ Drug abuse, dependence, and withdrawal, and modified SMQ Drug abuse, dependence, and withdrawal.

Figure 1 shows the distribution of adverse event cases retrieved at each step of data extraction for each drug group. The total number of cases initially retrieved per drug class was highest for benzodiazepines approved for any indication (67,584 cases), followed by Z-drugs (13,495 cases), trazodone (9,965 cases), DORAs (9,963 cases), benzodiazepines approved for insomnia (2,868 cases), doxepin (2,358 cases), and ramelteon (314 cases).

Of the 9,963 cases initially retrieved for DORAs, 2.6% (n = 262) were defined as modified SMQ Drug abuse, dependence, and withdrawal events. In contrast, 27.7% (n = 18,690), 23.0% (n = 659), and 15.3% (n = 2,063) of cases, respectively, fulfilled the criteria for the modified SMQ Drug abuse, dependence, and withdrawal for benzodiazepines approved for any indication, benzodiazepines approved for insomnia, and Z-drugs. For the non-scheduled drugs, the percentages of modified SMQ Drug abuse, dependence, and withdrawal events for trazodone (22.7%, n = 2,261), doxepin (22.3%, n = 525), and ramelteon (8.0%, n = 25) were higher than for DORAs.

### 3.1 Disproportionality analysis

**FIGURE 2:**
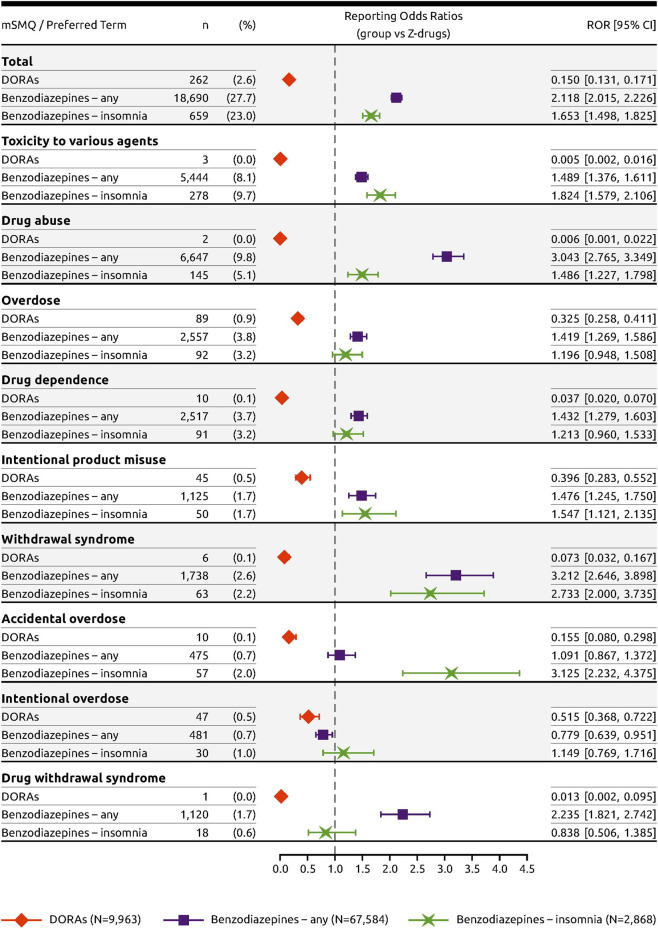
Forest plot of the RORs of DORAs and benzodiazepines with Z-drugs as reference.

Relative to Z-drugs, adverse event PTs within the modified SMQ Drug abuse, dependence, and withdrawal with frequency ≥1 in any of the groups included “toxicity to various agents,” “drug abuse,” “overdose,” “drug dependence,” “intentional product misuse,” “withdrawal syndrome,” “accidental overdose,” “intentional overdose,” and “drug withdrawal syndrome” (Figure 2). DORAs were associated with a low ROR value relative to Z-drugs overall (ROR = 0.150; 95% CI [0.131, 0.171], while the ROR value for benzodiazepines approved for any indication (ROR = 2.118; 95% CI [2.015, 2.226]) and benzodiazepines approved for insomnia (ROR = 1.653; 95% CI [1.498, 1.825]) were both >1 (Figure 2).

**FIGURE 3:**
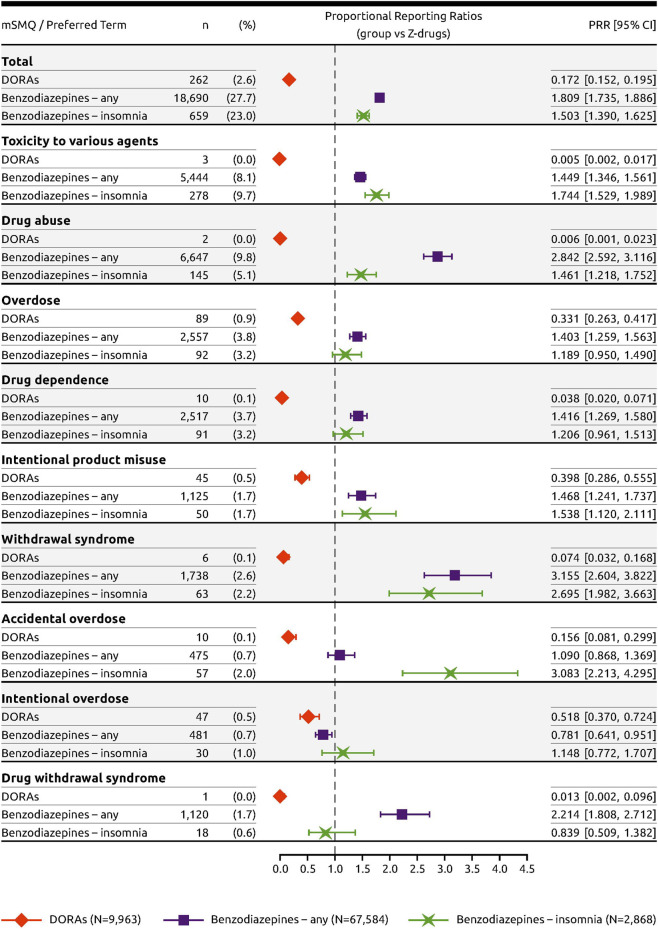
Forest plot of the PRRs of DORAs and benzodiazepines with Z-drugs as reference.

For all drug abuse PTs with a frequency threshold ≥1% in any of the drug groups, DORAs had significantly fewer cases reported in proportion to the Z-drugs (Figure 2). For these PTs, the RORs were low, ranging from 0.005 to 0.515, with none of the upper limit of the 95% CI crossing the parity with Z-drugs. In contrast, almost all RORs were >1 for both classes of benzodiazepines. For benzodiazepines approved for any indication, ROR values > 2 were identified for “drug abuse” (ROR = 3.043; 95% CI [2.765, 3.349]), “withdrawal syndrome” (ROR = 3.212; 95% CI [2.646, 3.898]), and “drug withdrawal syndrome” (ROR = 2.235; 95% CI [1.821, 2.742]). Likewise, for benzodiazepines approved for insomnia, ROR values >2 were identified for “withdrawal syndrome” (ROR = 2.733; 95% CI [2.000, 3.735]) and “accidental overdose” (ROR = 3.125; 95% CI [2.232, 4.375]). Similar results were obtained using the PRR to measure disproportionality (Figure 3).

**FIGURE 4:**
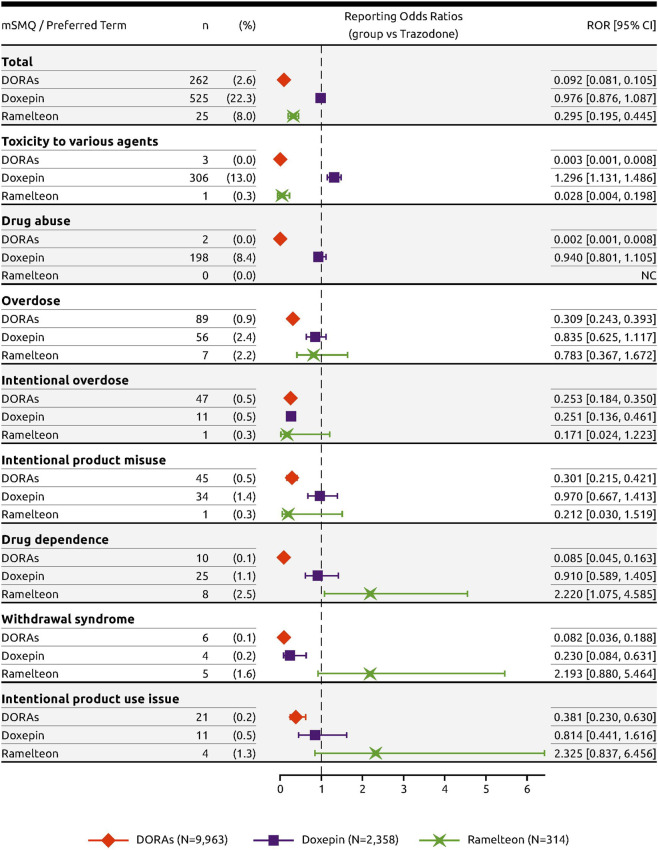
Forest plot of the RORs of DORAs, doxepin, and ramelteon with trazodone as reference.

Relative to trazodone, adverse event PTs within the modified SMQ Drug abuse, dependence, and withdrawal with frequency ≥1 in any of the groups included “toxicity to various agents,” “drug abuse,” “overdose,” “intentional overdose,” “intentional product misuse,” “drug dependence,” “withdrawal syndrome,” and “intentional product use issue” (Figure 4). Both DORAs (ROR = 0.092; 95% CI [0.081, 0.105]) and ramelteon (ROR = 0.295; 95% CI [0.195, 0.445]) were associated with a low ROR value relative to trazodone overall while the ROR value for doxepin (ROR = 0.976; 95% CI [0.876, 1.087]) was similar to that of trazodone (Figure 4).

**FIGURE 5:**
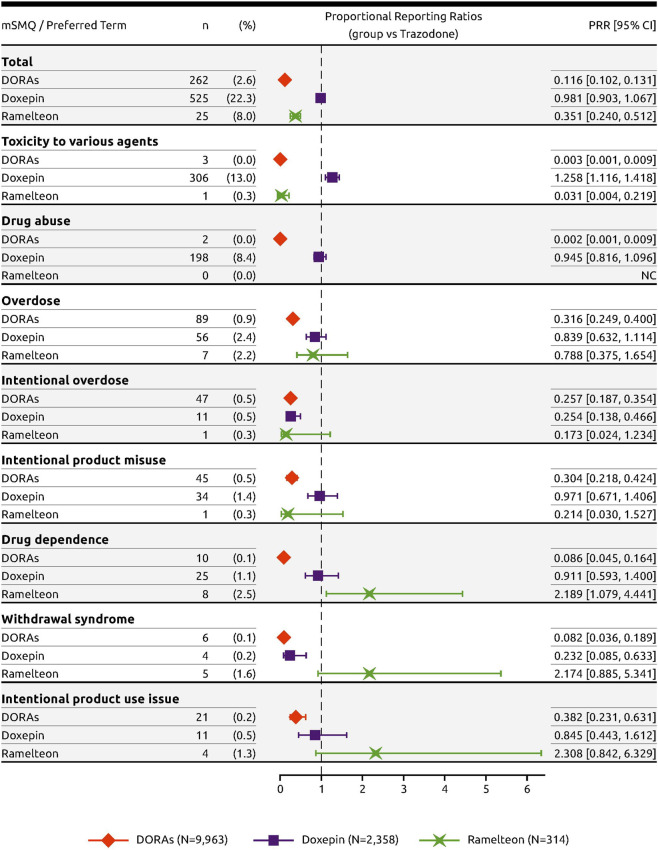
Forest plot of the PRRs of DORAs, doxepin, and ramelteon with trazodone as reference.

For all drug abuse PTs with a frequency threshold ≥1%, DORAs had significantly fewer cases reported in proportion to trazodone. For these PTs, the RORs were low, ranging from 0.002 to 0.381 (Figure 4). In contrast, ramelteon had three PTs with ROR values above 2: “drug dependence” (ROR = 2.220; 95% CI [1.075, 4.585]), “withdrawal syndrome” (ROR = 2.193; 95% CI [0.880, 5.464]), and “intentional product use issue” (ROR = 2.325; 95% CI [0.837, 6.456]), while doxepin had one: “toxicity to various agents” (ROR = 1.296; 95% CI [1.131, 1.486]). Similar results were obtained using the PRR to measure disproportionality (Figure 5).

## 4 Discussion

This study evaluated the drug abuse potential associated with commonly used hypnotics in a real-world pharmacovigilance study of commercially available drugs based on data from the FAERS database. As several of these widely prescribed medications for insomnia were approved in the 1980s under varying regulatory frameworks, comprehensive data on their abuse potential from large randomized clinical trials are lacking. In this context, the use of a large and unified real-world data source is of specific relevance. To our knowledge it is the most extensive analysis on the drug abuse potential of DORAs conducted to date after the market entrance of the DORA class in 2014, spanning a 10-year reporting period of pharmacovigilance experience.

Based on both ROR and PRR disproportionality analyses, our results clearly show that the DORA class had significantly lower odds of reporting for adverse events denoting drug abuse (as per the modified SMQ) when compared with both Z-drugs and trazodone as reference groups. Conversely, benzodiazepines, considered as a group or restricted to those approved for insomnia, were associated with the highest odds of abuse potential compared with Z-drugs. Our results are also consistent with the finding that, despite trazodone not being scheduled under the Controlled Substances Act, both Z-drugs and trazodone are associated with significant dependence and abuse potential (De Crescenzo et al., 2022; Zheng et al., 2022; Yu et al., 2025; Amari et al., 2022; Victorri-Vigneau et al., 2014). It should also be noted that we have been conservative with the DORA class by removing fewer cases in the modified SMQ Drug abuse, dependence, and withdrawal relative to the other drugs (i.e., not biased to the ROR and the PRR).

We were surprised to discover the large proportion of adverse events reported that were associated with drug abuse or dependence with the use of trazodone. While the data for trazodone does not specify whether patients receiving this medication were being treated off-label for insomnia or underlying depression or anxiety-related disorders, there is growing evidence that most of the trazodone prescribed in the US is in the service of improving sleep-related symptoms (Wickwire et al., 2024).

Trazodone was developed in the 1960s in Italy and was approved in the U.S. in 1981 as the first non-tricyclic antidepressant for the treatment of depression. It is commonly prescribed “off-label” to treat insomnia (Wong et al., 2020) contrary to current American Academy of Sleep Medicine guidelines (Sateia et al., 2017). Moreover, a recent systematic review and meta-analysis found that adverse effects associated with trazodone necessitated a careful assessment of its risk-to-benefit ratio (Kokkali et al., 2024). Two separate meta-analyses also showed limited efficacy for trazodone, which is associated with poor tolerability (De Crescenzo et al., 2022; Zheng et al., 2022). A recent analysis of the FAERS database focused on trazodone identified a range of significant adverse event signals that were not evident in package inserts and suggested precautions for overdosing and dose adjustments. Consistent with our analysis, drug abuse emerged as a new potential adverse event to be monitored. The highest number of A/E reports were associated with patients being treated for insomnia with only 1% of reports associated with patients identified with Major Depression (Yu et al., 2025).

Recent publications examining large databases employing a matched cohort design have documented a two-fold increase in falls and AEs associated with low-dose trazodone, relative to other medications used for insomnia treatment (Amari et al., 2022; Wickwire et al., 2024). Additionally, this retrospective evaluation of Medicare claims data demonstrated higher health-related costs associated with trazodone. Psychiatric and nervous system disorders were the most common reports, with increased numbers of reports associated with suicide, drug abuse, as well as cardiac and respiratory impairment. These findings highlight the potential dangers associated with routine use of trazodone, particularly in an elderly or primary care setting (Albanese et al., 2023).

The finding that ramelteon, a melatonin-receptor agonist, would be associated with features identified as drug abuse or dependence is also surprising. As an unscheduled product for insomnia, ramelteon is not typically thought of as being prone to abuse or dependence. In a human abuse potential study for ramelteon, there was no significant effect found for any of the subjective-rated measures related to potential for abuse (drug liking, street value). Furthermore, 79% of participants identified the highest dose of ramelteon as placebo (Johnson et al., 2006). The findings on the abuse potential of ramelteon are therefore hard to interpret given its mechanism of action (Neubauer, 2008).

In our study, benzodiazepines had the highest odds of abuse potential compared with Z-drugs. This finding is congruent with a post-marketing study that assessed the relative abuse liability of sedative-hypnotic drugs in a drug-addicted population, wherein benzodiazepines were associated with a higher risk of abuse compared with Z-drugs and trazodone (Jaffe et al., 2004). Benzodiazepines were also identified as one of the top 10 drug classes relating to drug overdose reported to FAERS from 2017 to 2021 (Ni et al., 2023). Although only five benzodiazepines are currently approved by the FDA for insomnia (estazolam, flurazepam, quazepam, temazepam, triazolam), sedation is a known side effect of benzodiazepines, and many are used off-label as hypnotics (Madari et al., 2021).

The mechanism of a benzodiazepine at the receptor site is most likely the major determinant of dependence and withdrawal. Benzodiazepines bind to the benzodiazepine-GABA receptor complex, enhancing the activity of GABA to hyperpolarize neurons, thereby increasing CNS inhibition. Chronic benzodiazepine treatment can lead to GABA downregulation. The abrupt discontinuation of benzodiazepines may then lead to an acute reduction in GABA, leading to a more excited and less inhibited CNS (American Psychiatric Association, 1990; Wofford, 2005).

Z-drugs likewise act via inhibition of specific sub-units of the GABA receptors in the brain and can induce tolerance, resulting in dose increase, physical and psychological dependence, craving and withdrawal symptoms. Most treatment guidelines recommend z-drugs only for short-term use in insomnia. Despite this, analysis of US Medicare claims data demonstrated that over 23% of recipients were receiving a prescribed insomnia medication with 30% receiving zolpidem and markedly increasing numbers receiving prescriptions for trazodone (Wong et al., 2020). A more recent analysis of the European Medicines Agency Database showed that over 275,000 adverse events were reported associated with z-drug use from 2003 to 2017, with intentional overdose and reported overdose rates between 14% and 30% (Schifano et al., 2019).

Hence, there may be considerable negative consequences for patients with chronic insomnia because of an expanded use of benzodiazepines and trazodone (despite the increased risk of abuse and the limited efficacy for trazodone) due to the Schedule IV placement of DORAs. In fact, DORAs have shown a positive benefit/risk profile, as acknowledged by recent European Sleep Research Society guidelines, which recommend daridorexant as the only drug after CBT-i for both short- and long-term treatment of chronic insomnia, with grade A evidence (RIemann et al., 2023). Additionally, there is now emerging evidence that DORA medications have been evaluated as adjunct therapy to improve sleep and decrease symptoms of withdrawal in patients undergoing medication-assisted therapy for opiate use disorder (Huhn et al., 2022). A recent retrospective review suggests that daridorexant, the most recently approved DORA medication, effectively targeted insomnia symptoms in patients with substance use disorder without adversely affecting safety (Di Nicola et al., 2025).

### 4.1 Study limitations

Although these findings provide a comprehensive perspective in the real-world evaluation of insomnia medicines as well as DORA-related drug abuse potential, the results of the present study should be interpreted considering some limitations. FAERS data are based on spontaneous reports; thus, the presence of duplicate reports, reporting errors, and high variability in the general quality and completeness of reported data should be considered. Reported events denoting drug abuse may only represent a partial, and possibly underrepresented, percentage of all drug abuse occurring in routine clinical use. The absence of data on the number of patients effectively treated with these drugs during the specified period (i.e., the denominator for calculating incidence fractions) precludes the calculation of incidence rates.

The identification of high rates of adverse events associated with abuse characteristics for the benzodiazepines is not surprising. Although there are 5 benzodiazepines approved for the treatment of insomnia it is understood that off-label prescribing of other benzodiazepines (e.g., lorazepam, clonazepam, alprazolam) may take place in the presence of comorbid features of anxiety or depression with insomnia disorder. By separating the “approved for insomnia” agents from the “unapproved for insomnia” group we attempted to better understand the risks associated with medications used primarily for insomnia disorder. Recognizing that overlap with “unapproved” agents might exist it seems unlikely that the 5 drugs indicated for insomnia disorder would be used for other conditions.

In any real-world comparison of different medications it is helpful to understand the incidence of a product’s use, identifying the denominator against which adverse events are measured. It is difficult to capture the extent or incidence of specific drug use for insomnia. Claims data sets from electronic health records (HER) can be prone to bias and errors arising from possible misclassification in data entry. This is often associated with the EHR data being used for purposes of medical billing, influencing provider behavior. This can be compounded by data propagation, a process through which a provider or medical assistants routinely “copy and paste” individual patient information without appropriate documentation of changes in the patient’s medical history, including use of medications (Kan Kim et al., 2023). Actual prescribing databases provided by pharmaceutical manufacturers are likewise subject to suspicion given the financial incentives associated with prescribing patterns.

Disproportionality analyses are commonly affected by biases such as the notoriety bias and confounding by indication (Cutroneo et al., 2024). The impact of notoriety bias (also known as stimulated reporting) on this analysis is acknowledged; however, considering the period covered by this study reflects the first approval and commercialization of the three DORAs, this potential bias has not favored the DORA class since the reporting of adverse events is typically enhanced in the initial years of marketing as compared with older drugs for which the safety profile has already been established (Di Nicola et al., 2025). Regarding the confounding by indication bias, implementing two analyses (i.e., all hypnotics regardless of indication and all hypnotics approved/used for insomnia) mitigates this issue. Given the complexity of the conditions, including comorbidities, caution should be applied when interpreting the results.

## 5 Conclusion

In conclusion, this study identified significantly fewer reported cases of real-world abuse, misuse, overdose, and other safety risks for DORAs compared with Z-drugs, the main Schedule IV hypnotics, as reflected by a significant disproportion in favor of the DORA class. Results were similar when DORAs were compared with the unscheduled hypnotic trazodone, suggesting that, among the various classes of products analyzed, the DORA class represent the safest option. As the placement of the DORA class into Schedule IV was based primarily on the results of HAP studies, the absence of nonclinical or clinical data suggestive of a risk of abuse or dependence among the class of DORAs should modify their risk versus benefit balance relative to other drugs used to treat insomnia. The categorization of DORAs as Schedule IV drugs therefore may overstate their abuse potential and create access and/or prescribing barriers for this class of drugs in the daily treatment of insomnia.

## Data Availability

Publicly available datasets were analyzed in this study. This data can be found here: FDA Adverse Event Reporting System.
